# Genetic evidence for sexual reproduction and multiple infections of Norway spruce cones by the rust fungus *Thekopsora areolata*

**DOI:** 10.1002/ece3.6466

**Published:** 2020-06-17

**Authors:** Hernán Capador, Berit Samils, Juha Kaitera, Åke Olson

**Affiliations:** ^1^ Department of Forest Mycology and Plant Pathology Swedish University of Agricultural Sciences Uppsala Sweden; ^2^ Natural Resources Institute Finland University of Oulu Oulu Finland

**Keywords:** aecia, cherry‐spruce rust, cone rust, forest pathology, life cycle, Norway spruce, *Picea abies*, seed orchard

## Abstract

Rust fungi are obligate parasites, of plants, with complex and in many cases poorly known life cycles which may include host alteration and up to five spore types with haploid, diploid, and dikaryotic nuclear stages. This study supports that *Thekopasora areolata*, the causal agent of cherry‐spruce rust in Norway spruce, is a macrocyclic heteroecious fungus with all five spore stages which uses two host plants *Prunus padus* and *Picea abies* to complete its life cycle. High genotypic diversity without population structure was found, which suggests predominantly sexual reproduction, random mating and a high gene flow within and between the populations in Fennoscandia. There was no evidence for an autoecious life cycle resulting from aeciospore infection of pistillate cones that would explain the previously reported rust epidemics without the alternate host. However, within cones and scales identical multilocus genotypes were repeatedly sampled which can be explained by vegetative growth of the fertilized mycelia or repeated mating of mycelium by spermatia of the same genotype. The high genotypic diversity within cones and haplotype inference show that each pistillate cone is infected by several basidiospores. This study provides genetic evidence for high gene flow, sexual reproduction, and multiple infections of Norway spruce cone by the rust fungus *T. areolata* which expands the general understanding of the biology of rust fungi.

## Graphical abstract

The biology and epidemiology of obligate parasites and in particular rust fungi are poorly understood since they are notorious difficult to study. The life cycle of rust fungi includes host alteration and up to five spore types with haploid, diploid, and dikaryotic nuclear stages which complicates the analyses of them. In this manuscript we show several feature and particularities of the rust fungi *Thekopsora areolata* which recently become a serious threat to seed production in Norway spruce (*Picea abies*). In our study, we used newly develop microsatellite markers to analyze the population structure and epidemiology; the study answers important questions of *T. areolata* biology, its reproduction, infection biology, and mode of spread in host tissue.

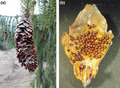

## 1 INTRODUCTION

Rust fungi are obligate parasites, of plants, that belong to the order Pucciniales of the phylum Basidiomycota. It is a large group with about 7,000 species belonging to this order. Rust fungi colonize plants from fern to higher plants and cause diseases of economic significance in agriculture, horticulture, and forestry. These fungi have complicated life cycles with up to five different spore stages. The life cycles often involve alternation between two unrelated host plant species to be complete (heteroecious life cycle). However, some species can complete their life cycle on a single host plant (autoecious life cycle). Although many rust species require two unrelated host plant species to complete their life cycles, rust species usually have a narrow and specific host range (Cummins & Hiratsuka, 2003). The different spore and life stages include haploid, diploid, and dikaryotic nuclear stages. The reproduction mode of different species of rust fungi varies from strict clonal to obligate sexual while many species utilize both sexual and asexual reproduction. The obligate nature and complex life cycles make rust fungi difficult to study, and therefore, the life cycle of many species is often poorly understood.

Conifers are a major component of boreal and alpine forests that cover large areas of northern hemisphere. These boreal forests are of great ecological and economic importance for many countries. Norway spruce [*Picea abies* (L.) Karst] is one of the most economically important conifer for the forest industry in Europe and among the most widely planted species. In Sweden and Finland, more than 200 and 100 million Norway spruce seedlings, respectively, are planted in managed forests annually (Haapanen, Jansson, Bräuner Nielsen, Steffenrem, & Stener, 2015; Himanen, 2016). About half of these seedlings originate from plant nurseries which use genetically improved seeds from seed orchards planted with superior plants from breeding programs. However, mass production of improved seeds for *P. abies* is usually lower than the demand (Haapanen et al., 2015), mainly due to irregular flowering and cone production between years and occurrence of pests, that is insects and fungal diseases (Kaitera, Hiltunen, Kauppila, Pitkäranta, & Hantula, 2014). In Finland, good seed crops have repeatedly suffered from cherry‐spruce rust caused by *Thekopsora areolata* (Fr.) Magnus (Kaitera, 2013), a rust fungus which grows in *P. abies* cones and shoots and reduces seed viability up to 10‐fold (Kaitera & Tillman‐Sutela, 2014).

**FIGURE 1:**
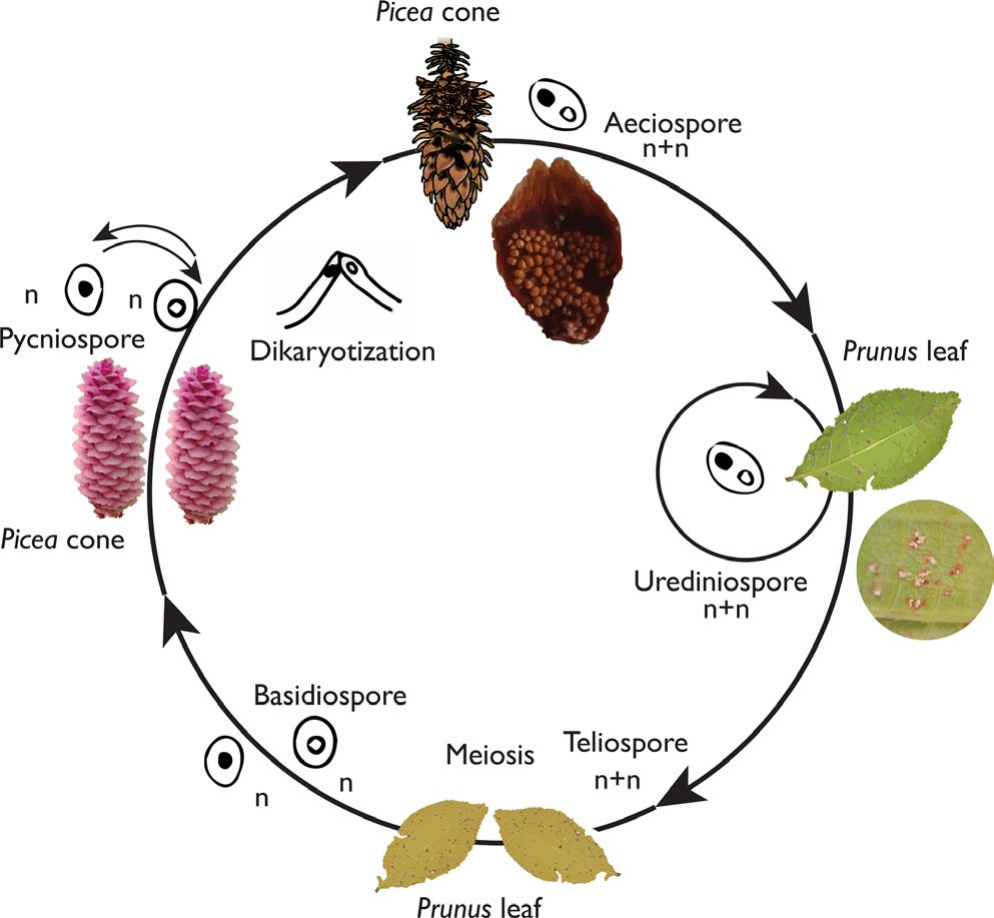
Life cycle of *Thekopsora areolata*.

**FIGURE 2:**
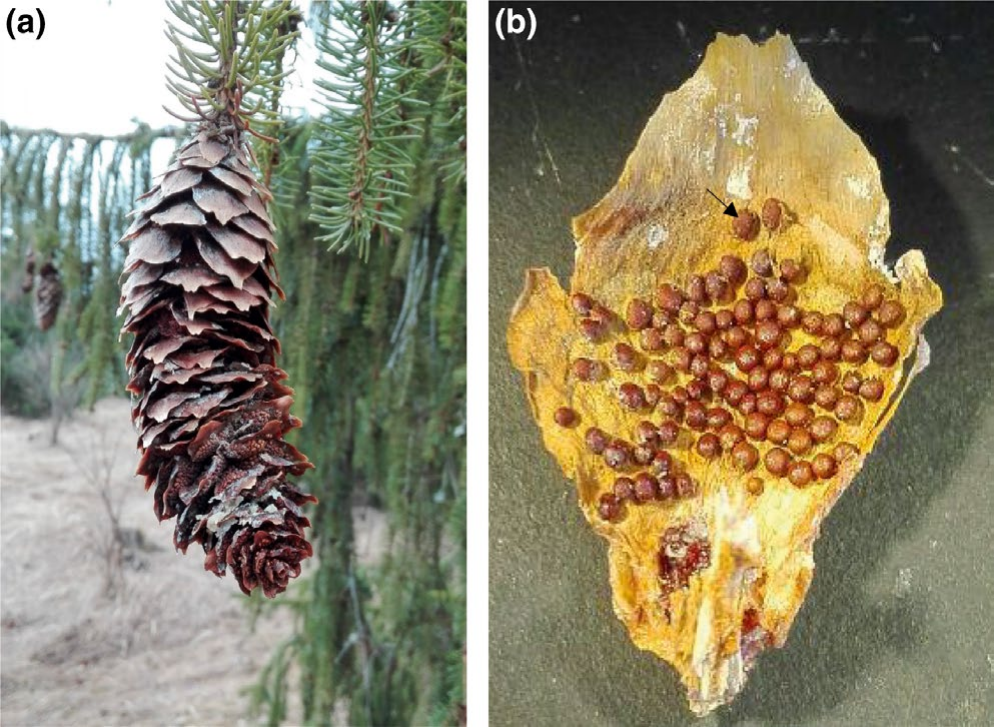
A *Picea abies* cone and a cone scale infected by *Thekopsora areolata*. (a) A diseased *P. abies* cone infected by *T. areolata*. (b) The rust grows throughout the scale of a cone forming aecia (arrow).

One of the main strategies to increase the production of improved seeds is by intensive seed orchard management (Haapanen et al., 2015). However, cherry‐spruce rust imposes a challenge in disease control due to the complexity of its life cycle and reproduction biology. *Thekopsora areolata* has a 2‐year long life cycle with five different spore stages and host alternation between *Picea* spp. and *Prunus* spp. (Figure 1) (Gäumann, 1959; Kuprevich & Tranzschel, 1957). In the spring, monokaryotic basidiospores infect *P. abies* pistillate cones, where mycelium grows through the axis of the developing cone and forms spermogonia composed of several flexuous hyphae and spermatia on the outer surface of scales. Rust spermatia and flexuous hyphae from genetically distinct individual fuse and develop into dikaryotic mycelium after anastomosis. The resulting heterokaryon will in turn form aecia, globoid structures composed of a thick‐walled peridium that bears inside chains of dikaryotic aeciospores. Aecia usually crowd the adaxial and often the abaxial surface of the majority of cone scales within months after fusion, but break and release spores in the spring the next few years (Figure 2) (Kaitera & Tillman‐Sutela, 2014). These aeciospores infect *Prunus* leaves, where uredinia with dikaryotic urediniospores are formed. Urediniospores reproduce clonally and can spread through re‐infection of *Prunus* leaves during the same season until autumn. Thereafter, the fungus overwinters in fallen *Prunus* leaves where the fungus develops telia and teliospores. Basidia and basidiospores will germinate from the overwintered teliospores as the result of karyogamy and meiosis, and basidiospores are disseminated in the spring to infect *Picea* (Gäumann, 1959).

A better understanding of the complex life cycle of *T. areolata* will provide new insights in the rust fungus' biology, which are essential for development of seed orchard management strategies. The loss of a part of the life cycle is a common repeated and independent event in rusts (Ono, 2002). The pine rust *Cronartium flaccidum* (Alb. & Schwein) G. Winter is heteroecious alternating between two‐needle pines and various herbaceous plants, while species of the *Peridermium* genus (syn. *Endocronartium*), *P. pini* (Willd.: Pers. Lév.) and *P. harknessii* (J.P. Moore), are autoecious rusts spreading from pine to pine (Hansen, Lewis, & Chastagner, 2018; Kaitera, Hiltunen, & Samils, 2012; Kaitera, Hiltunen, & Hantula, 2015). Today, *C. flaccidum* and *P. pini* are considered as two forms of the same species, *Cronartium pini* (Willd.) Jørst. (Hantula, Kasanen, Kaitera, & Moricca, 2002; Samils, Ihrmark, Kaitera, Stenlid, & Barklund, 2011), but in inoculation experiments aeciospores of the heteroecious *C. pini* (syn. *C. flaccidum)* have been shown to infect only alternate hosts and aeciospores of the autoecious *C. pini* (syn. *P. pini*) only *Pinus* spp. and not vice versa (Kaitera & Nuorteva, 2008). It has been hypothesized that short‐cycling in rusts might be a strategy in northern latitudes to survive in the absence of alternate hosts under harsh conditions (Savile, 1953). For *T. areolata,* it has been hypothesized that aeciospores from infected spruce cones might re‐infect spruce flowers or developing young cones directly, that is having an autoecious reproduction (Kaitera, 2013; Kaitera et al., 2014; Kaitera & Tillman‐Sutela, 2014). The hypothesis is based on the observation of severe rust damage without the presence of the alternate host *P. padus* (Kaitera, Tillman‐Sutela, & Kauppi, 2009). An autoecious life cycle would explain rust epidemics in remote areas in the north, where *Prunus* trees are either rare or lacking. If spruce infection with aeciospores was possible, it would mean that inoculum was often available close to the spruce flowers and developing cones. Infected cones remain attached in tree canopy for several years, and germination experiments have shown that aeciospores remain viable for at least 4 years (Kaitera & Tillman‐Sutela, 2014). In such a case, removal of all infected cones from a seed orchard would greatly reduce the risk of disease outbreaks and improve the health and quality of seeds. Another consequence would be that *T. areolata* would reproduce clonally on spruce in addition to the clonal reproduction with urediniospores on *Prunus* spp.

Direct studies of the reproduction mode in rust fungi are difficult, but indirect inferences can be made with help of molecular markers as shown in other fungal systems. For instance, the use of microsatellite or simple sequence repeat (SSR) markers with a correct sampling strategy can help to improve knowledge in many areas of fungal population biology (Dutech et al., 2007; Lim, Notley‐McRobb, Lim, & Carter, 2004) such as population differentiation (Ali et al., 2014), genotypic and genetic diversity (Barres, Dutech, Andrieux, Halkett, & Frey, 2012), heterozygosity and life‐history characteristics like mode of reproduction (Danies et al., 2014), and host selection (Berlin, Djurle, Samils, & Yuen, 2012). Molecular markers have also proved to be useful in population genetic studies of rusts causing diseases on trees, for example, on *Cronartium* spp. (Hamelin, Beaulieu, & Plourde, 1995; Samils et al., 2011), *Melampsora* spp. (Barres et al., 2008; Samils, Lagercrantz, Lascoux, & Gullberg, 2001), and *Austropuccinia psidii* (G. Winter) Beenken (Sandhu, Karaoglu, Zhang, & Park, 2016).

In this study, we investigate the reproduction mode and population genetic structure of the rust fungus *T. areolata* in Fennoscandia using a hierarchical sampling strategy and recently developed microsatellite markers (Capador, Samils, & Olson, 2018). We analyze the genetic and genotypic variation of *T. areolata* across different hierarchical levels in *P. abies* seed orchards and stands as well as genotype distribution in cones. Additionally, we apply haplotype inference methods to predict the most likely haplotype in the previous step of the life cycle. The combined results are used to make inferences on the mode of reproduction and epidemiology of *T. areolata*.

## 2 MATERIALS AND METHODS

### 2.1 Partially nested hierarchical sampling

**FIGURE 3:**
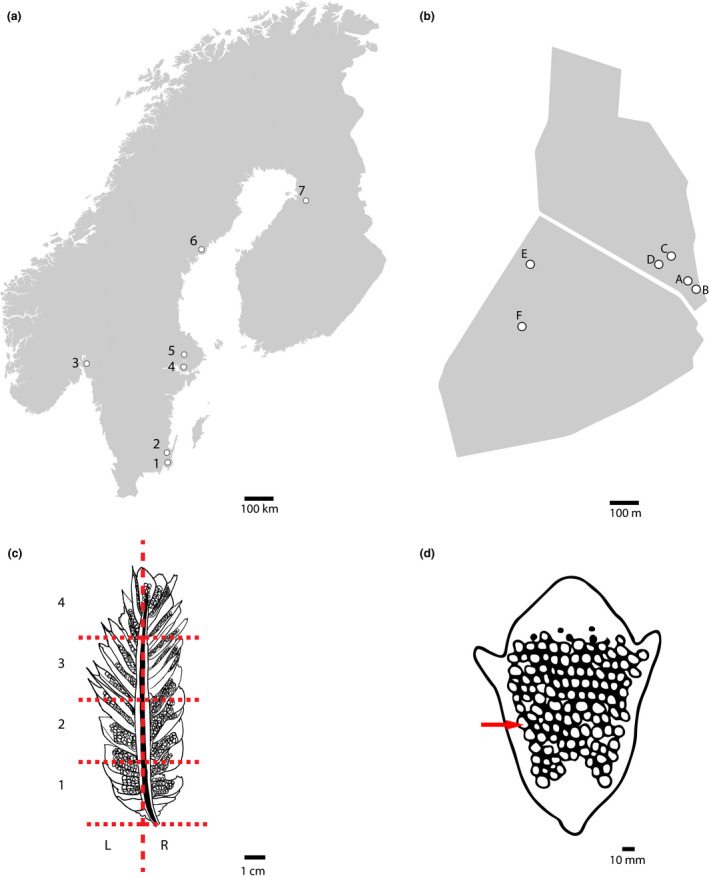
Partially nested sampling strategy. (a) Location level: 1. Bredinge (Öland, Sweden), 2. Söregärde (Småland, Sweden), 3. Ås (Akershus, Norway), 4. Ålbrunna (Uppland, Sweden), 5. Rörby (Uppland, Sweden), 6. Domsjöänget (Västerbotten, Sweden), and 7. Muhos (Northern Ostrobothnia, Finland). (b) Tree level: Individual trees sampled in Ålbrunna (A‐F). (c) Cone level: Cones were split longitudinally and infected scales were taken from 3 cm segments (red horizontal dashed lines) from both sides of the central axis (red vertical dashed line). (d) Scale level: Arrow indicates one aecium.

Location level: *Picea abies* cones with aecia were collected from 7 different locations in Sweden, Norway, and Finland (Figure 3a). At each location, 30 cones were collected, from which one scale with aecia per cone and one aecium per scale were randomly sampled. At tree level: A more extensive sampling was made at the seed orchard in Ålbrunna (Sweden), where 100 cones with aecia were sampled from 6 different trees at a distance of ca. 20 m–600 m from each other (Figure 3b). From each cone, one scale with aecia and one aecium per scale were randomly sampled (Figure 3b). At cone level: 10 cones with aecia were randomly sampled from two locations in Sweden (a‐1 and a‐4) and split longitudinally to select 10 scales across each cone, from which 10 aecia per scale were randomly sampled (100 aecia per cone) (Figure 3c). At scale level: 3 individual scales with aecia from cones from two different locations in Sweden (a‐1 and a‐4) were thoroughly sampled (ca. 40 aecia per scale; Figure 3d).

### 2.2 Single aecium genotyping

Cone scales with aecia were dipped for 45 min in a solution of 30% H_2_O_2_, with a drop of Tween 80 and rinsed three times in deionized water. Thereafter, a single aecium was picked with the help of a hooked needle. Because an aecium has been shown to contain a bulk of genetically identical dikaryotic aeciospores, each aecium was treated as one individual (Capador et al., 2018; Rodriguez‐Algaba, Sørensen, Labouriau, Justesen, & Hovmøller, 2017). Rodriguez‐Algaba et al., 2017). DNA was extracted from each aecium following the protocol of Capador et al. (2018). The samples were genotyped with 8 polymorphic microsatellites markers; Tha9, Tha61, Tha91, Tha92, Tha96, Tha105, Tha136, and Tha137, and microsatellite amplification and scoring was performed as formerly described (Capador et al., 2018) except for a subset of samples which were analyzed with DreamTaq (Thermo Fisher) instead of PIR00 (Sigma).

### 2.3 Data filtering

Due to the partly‐nested sampling strategy and to prevent biases caused by uneven population sizes, the dataset (in GenAlEx format) was curated by removing uninformative loci (minor allele frequency < 5%) and individuals with missing data in more than one loci. Before further analyses, the dataset was divided in four smaller datasets according to its hierarchical level (Figure 3) using the R package poppr 2.8.2 (Kamvar, Tabima, & Grünwald, 2014). At the location level, a maximum of 30 cones per location and one individual per cone was included. At the tree level, 100 cones in Ålbrunna were included with a maximum of one aecium per cone. At the cone level, only those cones with 100 aecia sampled per cone were included. At the scale level, each scale genotyped thoroughly was analyzed separately. Tree, cone, and scale levels were curated separately for uninformative loci (minor allele frequency < 10%) and missing data to improve the accuracy of clonality estimates.

### 2.4 Genotypic diversity

Genotypic diversity and population structure estimates were calculated at each hierarchical level using the R package poppr 2.8.2 (Kamvar et al., 2014). Genotypic diversity was estimated using the Shannon‐Wiener diversity index (H; Shannon, 2001) which gives the number of multilocus genotypes (MLGs) observed in relation to the number of samples analyzed and with the corrected Simpson index (λ; Simpson, 1949). The function Psex was used to estimate the probability of finding one MLG multiple times by chance (and not by clonal reproduction) at the location level according to Parks, and Werth, (1993) and Arnaud‐Haond, Duarte, Alberto, and Serrão (2007).

### 2.5 Clonality

The standardized Index of Association (rbarD) was used as a tool to detect clonal reproduction across hierarchical levels in R package poppr 2.8.2 (Kamvar et al., 2014). rbarD estimates the degree of linkage between markers and tests to what extent individuals that are the same at one locus are more likely than random to be the same at other loci (Agapow & Burt, 2001). *p* values for rbarD (P.rbarD) were computed based on a 999 one‐side permutation test (Kamvar et al., 2014). Furthermore, a conservative Psex value was calculated in RClone 1.0.2 to account for possible departures from Hardy–Weinberg equilibrium (Arnaud‐Haond et al., 2007).

### 2.6 Population structure

Population differentiation at all hierarchical levels was tested with analysis of molecular variance (AMOVA) (Excoffier, Smouse, & Quattro, 1992). The differentiation was calculated based on the Euclidean distance matrix at each level, and variation within samples could be interpreted as heterozygosity, because it measured differentiation between alleles at each loci across each sample. *p* values were computed based on 999 permutations (Excoffier et al., 1992; Kamvar, Brooks, & Grünwald, 2015; Kamvar et al., 2014). To identify possible population structure, discriminant analysis of principal components (DAPC) was used at the location hierarchical level. DAPC combined a principal component analysis (PCA) with a discriminant analysis (DA) to weight variability between previously defined populations more than variability within them (Jombart, Devillard, & Balloux, 2010). Additionally, Fst was calculated with GenAlEx (Peakall & Smouse, 2006) to estimate pairwise differences between populations. To analyze further the population structure, we used the program STRUCTURE 2.3.4 that uses a Bayesian approach to assign individuals into groups (clusters) based on genetic similarity (Pritchard, Stephens, & Donnelly, 2000). STRUCTURE was run by varying the number of clusters (K) from 2 to 5. The admixture model assuming no linkage between the loci and without a priori information on populations was applied. For each K, we made 3 repeated simulations with a burn‐in period of 500,000 iterations of the Markov Monte Carlo Chain (MCMC) and a run length of 500,000 MCMC iterations. Results were compiled using Structure Harvester (Earl & von Holdt, 2012) and bar plots constructed using the program Distruct (Rosenberg, 2004).

### 2.7 Haplotype inference

Haplotypes were inferred using the Excoffier‐Laval‐Balding (ELB) algorithm implemented in Arlequin 3.5 (Excoffier & Lischer, 2010) for the cone and scale hierarchical levels. This algorithm reconstructed unknown gametic phases by performing iterations and phase updates made on the basis of haplotype frequencies in the population and linkage between neighboring loci. In this case, the order of the markers was unknown, but they were assumed to be largely unlinked (Capador et al., 2018); therefore, any given order was irrelevant in this case. The value *γ* = 0.1 was used since very low recombination was expected in the analyzed hierarchical levels (see discussion), *α* = 0.01 was used to account for the possibility of finding a yet unobserved haplotype, and parameter *ε* = 0.1 was used for microsatellite data. The resulting best haplotypes for each individual were formatted with and visualized in R.

## 3 RESULTS

In total, 951 single aecia were collected from 7 locations in Fennoscandia and across 4 hierarchical levels; location, tree, cone, and scale. A strict filtering to remove genotypes with missing data in more than one loci resulted in a total of 520 successfully genotyped individuals with 7 polymorphic microsatellite markers (marker Tha109 with an MAF < 0.5% was considered uninformative). For analyses of the population structure, 30 cones were collected from seven locations (populations) in Fennoscandia, and samples consisted of one aecium per cone. The genotyping success was between 30% and 83% (Table 1). The mean number of alleles (Ne) per population varied between 2.75 and 4.25 with the highest Ne and mean allele richness (Ar), as well as the highest number of private alleles (N pri) being found in the Söregärde population (Sweden; Table 1). The lowest Ne, Ar, and N pri were found in the Domsjöänget population (Sweden; Table 1). The Simpsons diversity index (*λ*) and Shannon‐Wiener diversity index (H) were highest in Muhos (Finland) and lowest in Ås (Norway) while gene diversity (Hexp) was highest in Söregärde and lowest in Bredinge (Sweden; Table 1).

**TABLE 1:** Population diversity parameters for the populations of *Thekopsora areolata*

Parameters	Ålbrunna (SE)	Bredinge (SE)	Rörby (SE)	Söregärde (SE)	Domsjöänget (SE)	Ås (NO)	Muhos (FI)	Total Fennoscandia
*N*	22	18	12	14	11	10	25	112
MLG	22	17	12	14	11	10	25	107
Ne	3.88	3.250	3.88	4.250	2.75	3.00	4.125	6.875
*N* pri	3	2	3	6	1	3	3	NA
Ar	3.10	2.69	3.42	3.54	2.52	2.89	3.12	
H	3.091	2.813	2.485	2.639	2.398	2.303	3.219	4.644
*λ*	0.955	0.938	0.917	0.929	0.909	0.900	0.960	0.990
Hexp	0.386	0.333	0.460	0.479	0.370	0.391	0.392	0.401
rbarD	−0.033	0.028	0.015	−0.016	−0.001	−0.029	0.011	−0.004
p.rbarD	.946	.155	.352	.677	.466	.725	.312	.662

Abbreviations: Ar, Mean allele richness; FI, Finland; H, Shannon‐Wiener Index of MLG diversity (Shannon, 2001); Hexp, Nei's unbiased gene diversity; MLG, multilocus genotypes; *N* pri, number of private alleles; *N*, number of individuals; Ne, mean number of alleles;NA, Not analysed; NO, Norway; p.rbarD, *p*‐value for rbarD; rbarD, standardized index of association; SE, Sweden; *λ*, Simpson's index.

### 3.1 Random mating test for clonality

The number of MLGs equaled the number of individuals in all populations except in Bredinge where two individuals had the same MLG. In total, only three MLGs were sampled multiple times over the whole dataset (Table 1). To test the probability that these identical MLGs originated from distinct sexual events, and thus, were different genets and not clones/ramets of the same MLG, a test was performed using a conservative Psex estimation. Test results showed that only MLG 47 (found once in Ålbrunna, twice in Bredinge and once Söregärde) had a low probability (*p* = 3.3e^−10^) to be found by chance four times, while the other two are most likely a result of common alleles. To estimate the mode of reproduction of *T. areolata* and the probability of random mating, rbarD was calculated and the probability of observing its value was estimated with 999 permutations (Table 1). The rbarD values were low for all populations, and the *p* values were nonsignificant (Table 1). In all locations, the hypothesis of random mating and no linkage between markers was accepted.

### 3.2 Population structure and variation

**FIGURE 4:**
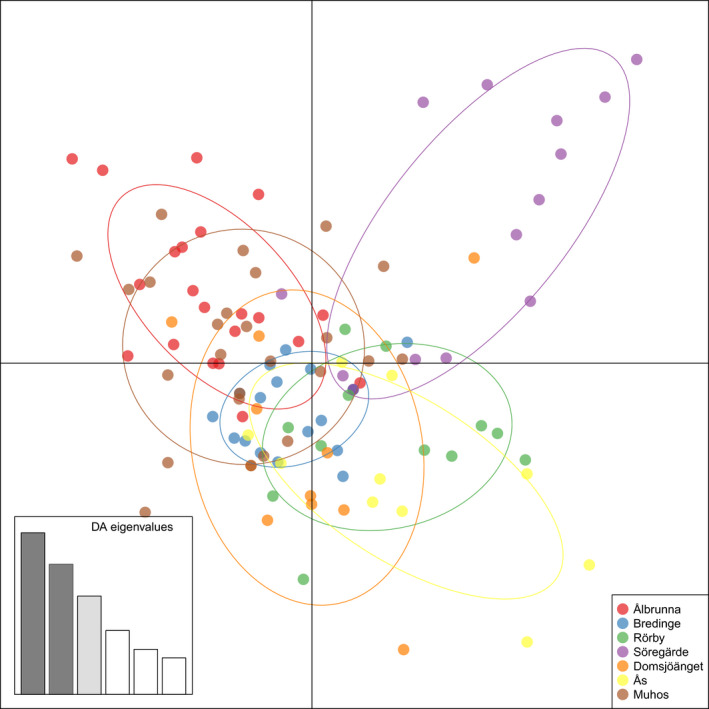
Discriminate analysis of principle components of *Thekopsora areolata*. Samples from each location are marked with distinct colors.

Of the total genetic diversity, most of the variation was attributed to the aecium (83%) and between aecia (16%) while location and country explained <1% of the variation found (Table 2). The results of AMOVA indicated that population differentiation was insignificant between countries or locations (*p* > .05). To reveal any structure of *T. areolata* populations at country and location level, a discriminate analysis of principle components (DAPC) was performed. Samples from different locations and countries overlapped each other with a small separation between them (Figure 4). As an exception, the Söregärde population differed slightly from the other populations. In addition, Pairwise Fst values were calculated to further analyze the differentiation between *T. areolata* populations from different locations (Table S1) as well as a Bayesian cluster analysis using software STRUCTURE (Figure S1) but no significant differences were found.

**TABLE 2:** The distribution of variance within and between locations in *Thekopsora areolata*

Hierarchical level	Variance (%)	*p* value	Phi value
Between countries	−0.149	.559	−0.001
Between locations, within country	0.672	.186	0.006
Between aecia, within location	16.384	.001**	0.165
Within samples	83.092	.001**	0.169

** Significant at *p* < .001.

### 3.3 Genotype and diversity parameters for tree, cone, and scale

The aecial samples from different cones from six trees in Ålbrunna showed high genotypic diversity, and the number of samples equaled the number of MLGs found in all trees (Table 3). The Simpsons diversity index (*λ*) and Shannon‐Wiener MLG diversity (H) ranged from 1.1 to 2.9 and 0.67 to 0.94, respectively, reflecting the increasing number of samples. The gene diversity was highest in the trees AL‐A and AL‐D (Hexp = 0.4) and lowest in the trees AL‐E and AL‐F. Contrastingly, at the cone and scale level identical MLGs were found repeatedly within the cones and scales (Table 3). Cone BR_202 contained three MLGs among the 14 aecia sampled (Table 3). However, in cone Br_231 each sampled aecia had a unique MLG (Table 3). In general, there was large variation in the number of MLGs per cone but in the scales the number of different MLGs was low (Table 3). To estimate the mode of reproduction of *T. areolata* and the probability of random mating at the tree, cone, and scale hierarchical levels, rbarD was calculated (Table 3). In five of the trees in Ålbrunna, the null hypothesis of random mating and no linkage between markers was accepted while in tree AL‐A a low but significant (*p* = .046) linkage among markers was found (Table 3). For the cones, the null hypothesis was rejected (*p* < .01) at two out of ten cones and at both scales analyzed (Table 3).

**TABLE 3:** Genotypes and population diversity parameters for the samples of *Thekopsora areolata* at tree, cone, and scale levels

Hierarchical level	*N*	MLGs	*H*	*λ*	Hexp	rbarD	p.rbarD
Tree							
AL_A	6	6	1.792	0.833	0.403	0.177	.046*
AL_B	3	3	1.099	0.667	0.383	−0.346	.999
AL_C	8	8	2.079	0.875	0.394	0.006	.409
AL_D	6	6	1.792	0.833	0.407	−0.079	.884
AL_E	18	18	2.890	0.944	0.356	−0.039	.911
AL_F	17	17	2.833	0.941	0.367	0.003	.419
Cone							
AL_6	20	11	2.398	0.909	0.403	0.087	.060
AL_37	12	10	2.303	0.900	0.358	−0.051	.867
AL_46	20	9	2.197	0.889	0.381	0.319	.001**
AL_69	23	10	2.303	0.900	0.413	0.084	.060
AL_89	19	10	2.303	0.900	0.247	−0.019	.551
BR_202	14	3	1.099	0.667	0.248	0.250	.179
BR_213	24	15	2.708	0.933	0.334	−0.016	.650
BR_231	18	18	2.890	0.944	0.413	0.035	.149
BR_285	33	18	2.890	0.944	0.376	0.020	.234
BR_255	20	12	2.485	0.917	0.304	0.125	.035*
Scale							
AL_88_1	26	4	0.484	0.213	0.237	0.669	.001**
AL_14_1	24	2	0.173	0.080	0.152	NA	NA
BR_221_1	18	6	1.303	0.630	0.107	0.399	.001**

Abbreviations: H, Shannon‐Wiener Index of MLG diversity (Shannon, 2001); Hexp, Nei's unbiased gene diversity; MLG, Number of multilocus genotypes; *N*, number of samples; NA, Not analysed; p.rbarD, *p*‐value for rbarD; rbarD, standardized index of association; *λ*, Simpson's index.

* Significant at *p* < .05.

** Significant at *p* < .001.

The AMOVA indicates that no significant variation was found between trees but variation occurred between aecia within trees (19%) and within aecia (82%, *p* < .001; Table 4). However, at the cone level, 19% (*p* < .001) of the variation could be attributed between cones and 83% (*p* < .001) within aecia while <1% of the variation was found between aecia within cones (Table 4).

**TABLE 4:** The distribution of variance within and between tree and cone hierarchical levels in *Thekopsora areolata*

Hierarchical level	Variance (%)	*p* value	Phi value
Between trees, within Location	−0.8	.710	−0.007
Between aecia, within trees	18.7	.001**	0.186
Within aecia	82.1	.001**	0.179
Clone corrected at cone level^a^			
Between cones, within location	18.7	.001**	0.187
Between aecia, within cone	−1.5	.691	−0.018
Within aecia	82.5	.001**	0.174

^a^At this level, only cones with more than 100 aecia genotyped were included.

** Significant at *p* < .001.

### 3.4 Haplotype inference and pedigree analysis within cones and scales

**FIGURE 5:**
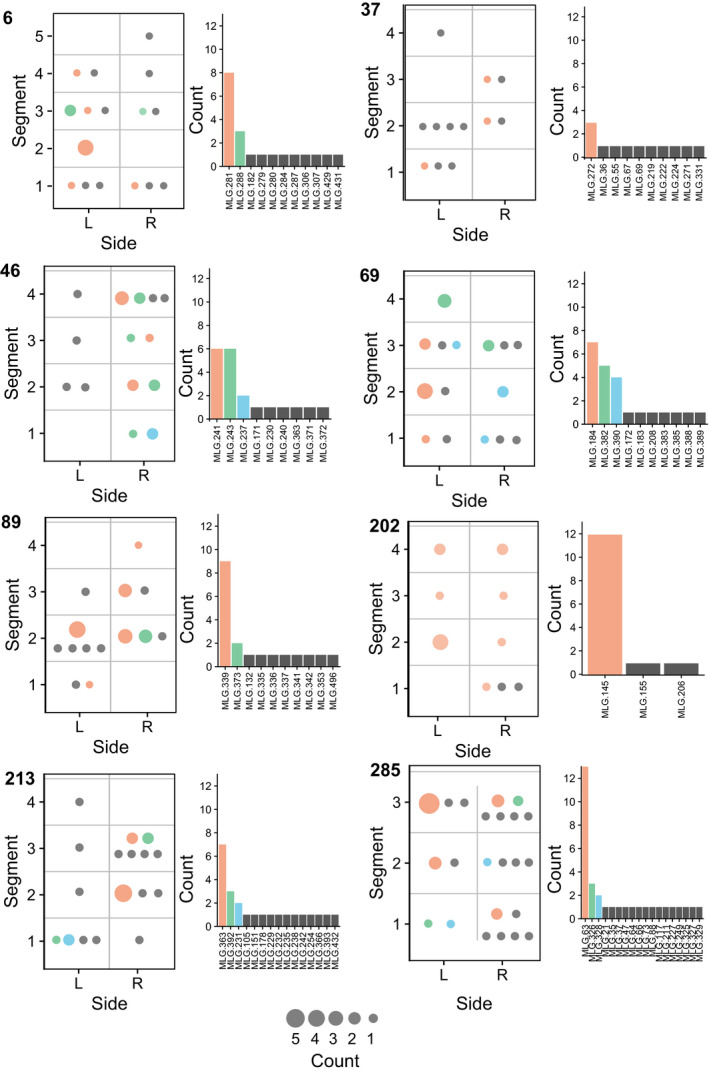
Frequency and distribution of MLGs across cones. Only samples with known location in the cone are included in figure. L and R represent samples from left and right side of the cone, and the segment represents the longitudinal position in the cone. For each cone, MLGs found more than once are presented in specific colors (the same color represents the same MLG in both the circles and bars, within one cone). The size of the circles represents the number of individuals with identical MLG. Gray circles represent MLGs found only once.

**FIGURE 6:**
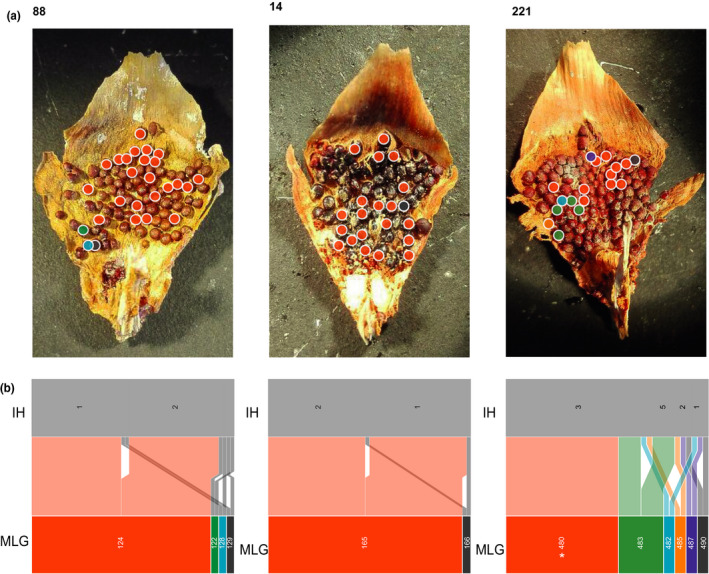
Fine scale genotyping and haplotype inference of *Thekopsora areolata* in three different scales. (a) Location of genotyped aecia within the scale. (b) Haplotype inference pedigree for the genotyped aecia. IH, inferred haplotype, MLG, multilocus Genotype. The color of the circles over the aecium in (a) corresponds to the color of the MLG in b). White asterisks denote MLGs homozygote for all loci.

At the cone and scale levels repeated MLGs were usually dominant in number (Figures 5 and 6). Most cones were dominated by one to three MLGs (Figure 5). The repeatedly sampled MLGs were usually located on more than one scale and throughout the cone (Figure 5). The samples from cone BR_202 represented almost exclusively (12 out of 14) by MLG 145 (Figure 5). The number of MLGs per scale for samples AL_88_1, AL_14_1, and BR_221_1 were 4, 2, and 6, respectively (Table 3). Two of the scales AL_88_1 and AL_14_1 had one dominating MLG, while in BR_221_1 two of the MLGs occurred several times (Figure 6a). Aecia of the dominant MLGs were spatially clustered, which also was true for the unique MLGs (Figure 6a).

**FIGURE 7:**
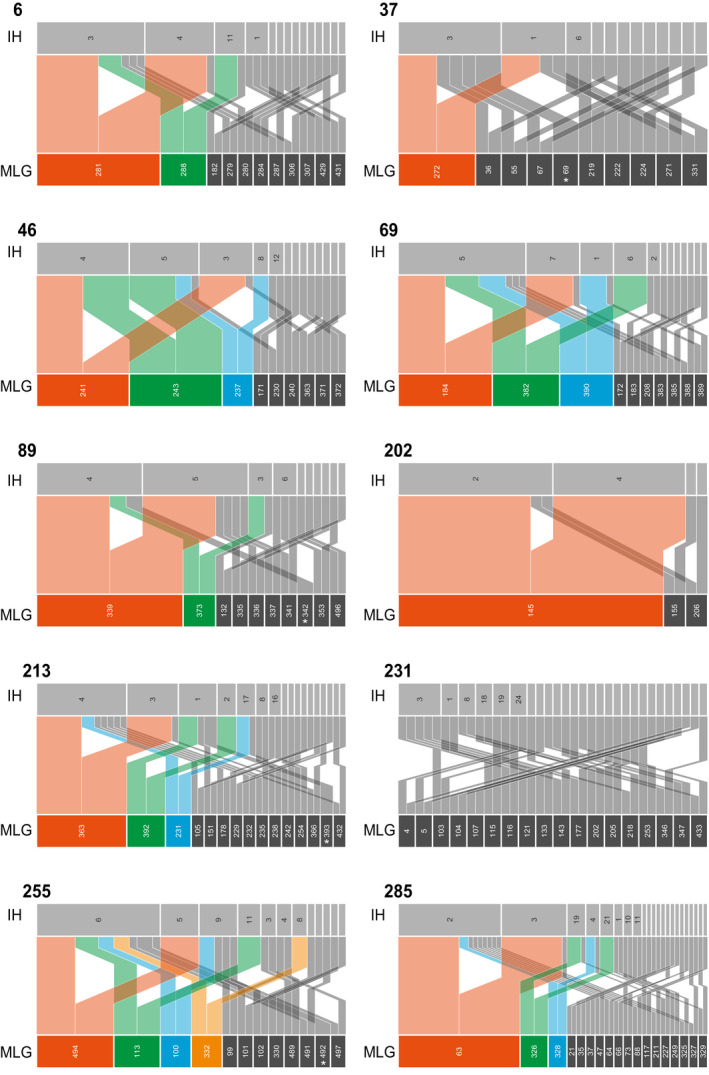
MLG and haplotype inference pedigrees in ten different cones. Haplotype inference was used to infer the haplotypes (IH) of each multilocus genotype (MLG) at the cone level. In color, MLGs repeated more than once in each cone, White asterisks denote MLGs homozygote for all loci.

Haplotype inference was used at the cone and scale level to trace back the most likely haploid genotypes (i.e., haplotypes) of the homokaryotic basidiospores that colonized the cone, and their probable mating events resulting in the observed heterokaryotic aecia. According to the haplotype inference, the dominant aeciospore MLG shared haplotypes with some of the other MLG in the cone in several cases (Figure 7, e.g., AL_69, AL_89, BR_202, BR_231). For some other cones, less haplotypes were dominant, and the present MLGs seemed to have arisen through combinations with single, less represented haplotypes (Figure 7: AL_6, AL_37, BR_255).

According to their predicted haplotypes, fertilization events between few haploid individuals could explain the genotypic diversity observed. In two of the scales just one mating event at each scale gave rise to dominating aecia (Figure 6b). In scale BR_221_1, the most frequent MLG was monomorphic at all loci (Figure 6, scale 221, MLG 480). The MLGs 342, 393, and 492 in the cones AL_88, BR_213, and BR_255, respectively, were also monomorphic at all loci according to the haplotype inference (Figure 7).

## 4 DISCUSSION

Details of the reproduction and epidemiology of many rust fungi are lacking, mainly due to the difficulties to study them because of their biotrophic nature and complex life cycle. In this study, we used population genetics tools to clarify features of the life cycle of *Thekopsora areolata*. We provided genetic evidence for high genotypic diversity, sexual reproduction, and multiple infection of pistillate cones of Norway spruce by *T. areolata* as well as detailed information of spread of fungal genotypes within cones and scales.

The rust *T. areolata* represents one population in Fennoscandia, with no evidence for any significant substructure or genetic differentiation between any of the populations analyzed. There is a large overlap between the populations in the DAPC plot, although the Swedish population Söregärde showed some difference from the other populations. However, pairwise F_st_ analysis did not support differentiation between the Söregärde population and any of the other populations in Fennoscandia. The difference found in the population from Söregärde can be explained by the high number of private alleles unique to this population. The reason why this population has higher number of private alleles than the other populations analyzed remains unanswered and needs additional studies to be resolved. The lack of population structure is likely to be maintained through a high level of gene flow between the populations, which is not surprising due to the nature of urediniospores as robust spores capable of dispersing long distances, even across continents (Brown, 2002). One similar example is *Melampsora epitea* Thümen on willows which is genetically highly diverse with small geographic differentiation in Sweden, probably due to high dispersal ability of urediniospores (Samils, Stepien, Lagercrantz, Lascoux, & Gullberg, 2001). Likewise, populations of *M. larici‐populina* Kleb. from European poplars show low genetic differentiation (Barres et al., 2008). In addition to urediniospores, aeciospores can be disseminated long distances leading to low geographical differentiation among *C. pini* populations in Finland, Sweden, and southern Europe (Hantula et al., 2002; Hantula, Niemi, Kaitera, Jalkanen, & Kurkela, 1998; Samils et al., 2011). Furthermore, the wide distribution of the both hosts of *T. areolata* in northern Europe increases the chances for long‐distance dispersed spores to infect susceptible *Prunus* and augments genotypic diversity, since sexual reproduction and recombination occur on this host.

At each seed orchards and stands, the number of MLGs equaled the number of samples in all populations except in Bredinge (Sweden) where one MLG was sampled twice. In addition, two more MLGs were found in more than one population. Analyses of the likelihood of finding the same genotype twice suggest that MLG 47 was not likely to be found by chance, and this suggests that MLG 47 is a true clone (Arnaud‐Haond et al., 2007). If correct, this result would imply that aeciospores of *T. areolata* are able to infect directly from pine to pine without passing the alternate host as well as spread over large distances since clone MLG 47 was found in three different seed orchards. However, further studies are needed to confirm this single case. It cannot be excluded that individuals within MLG 47 are not true clone mates but appear to be due to limited resolution of the markers used.

The high genotypic diversity, lack of population structure, and the low level of genetic linkage between markers were found in this study are consistent with random mating, suggesting that sexual reproduction is regularly occurring in *T. areolata* in Fennoscandia. In other rusts like *M. larici‐populina*, similar evidence has been found on populations known to reproduce sexually (Barres et al., 2012), where sexually derived basidiospores with unique genotypes are the main drivers of genotypic diversity, and they are also responsible for the observed linkage equilibrium (Rodriguez‐Algaba, Walter, Sørensen, Hovmøller, & Justesen, 2014).

In the cones, the genotypic diversity was lower with both unique and identical MLGs found within the same cone, and deviation from random mating was observed in a few cases even after clone correction (Table 4). Furthermore, we showed that there is usually one or a few dominant genotypes per cone. The dominating genotypes are present on different scales both along the central axis as well as on neighboring scales at the same level (Figure 5). These genotypes often share haplotypes as indication of family relationships in the same cone (Figure 7), which corroborates the deviation from random mating after clone correction. This pattern in MLG distribution sheds light into the reproduction mode of *T. areolata*: Firstly, usually more than two haplotypes per cone were inferred (Figure 7) indicating that multiple basidiospore infections can take place in the same cone. However, as only a few dominant MLGs were found throughout the cones, the basidiospore infections were local and vegetative spreading occurred only after the dikaryotization event had occurred. This is in line with Kuprevich and Tranzschel (1957), who reported that mycelium of *T. areolata* grows through the axis of the cone. Secondly, only a few genotypes dominated in scales and shared haplotypes with less common MLGs (Figure 6). The pattern we observed in cones and scales was similar to other rust pathosystems. In *Cronartium*, many aecia within lesions had different MLGs (Kasanen, Kaitera, & Hantula, 2000; Samils et al., 2011), and diversity of *Puccinia graminis* Pers., *P. coronata* Corda, and *P. striiformis* var. *tritici* Westend was high within aecial clusters in *Berberis vulgaris* L. (Berlin, Samils, & Andersson, 2017; Rodriguez‐Algaba et al., 2017). A study of the white pine blister rust, *Cronartium ribicola* show that 70–75% of the genetic variability is found between aecia within cankers while the rest is found between canker within site or between sites (Hamelin, 1996). It has been hypothesized that the high variability is due to single different cross‐fertilization events (fusion of spermatia or dikaryotization) in the same spermogonium. Berlin et al. (2017) showed that MLGs at each aecial cluster of *Puccinia* shared at least one haplotype. This strengthens the hypothesis of aecium formation due to single cross‐fertilizations between different flexuous hyphae from the same spermogonium (identical haploid genotype) and different spermatia (different haploid genotypes), resulting in different MLGs within the same aecial cluster (Berlin et al., 2017). However, this study showed that even within scales and cones, aecia shared more than one common haplotype, which implied spermatization with spermatia originating from more than one different spermogonia.

Most of the aecia sampled were heterozygotic; however, a few samples diverged from this pattern by being homozygous for all marker loci, which could be indicative of self‐fertilization (Figure 6, scale 221, MLG 480). However, the relative few markers used in this study suggest that the probability to find homozygous genotypes by chance with the allele frequencies present in Ålbrunna and Bredinge is high (*p* > .05). In general, it has been assumed that cross‐fertilization is needed for dikaryotization and that insects play a key role on the transport of spermatia to cross‐fertilize spermogonia (Naef, Roy, Kaiser, & Honegger, 2002). This has been supported by experiments in rust fungi *Uromyces pisi* (Pers.) Schrot, where insects were attracted by the honeydew matrix (in which the spermatia are embedded) and were required for the formation of the dikaryon (Pfunder & Roy, 2000). Additionally, it has been shown that either presence of insects or manual cross‐fertilization among different spermogonia resulted in successful aecia formation, while caged and self‐fertilized spermogonia formed a significantly lower number of aecia (Pfunder & Roy, 2000). We found identical homozygous MLGs in four cones and one scale analyzed, which suggests that the homozygotic MLGs in *T. areolata* could be a result of self‐fertilization between genetically identical spermatia of the same genet or lack of resolution due to the low number of SSR markers and the uneven allele distribution. This interesting observation needs further investigation to confirm or discarded the presences of self‐fertilization in *T. areolata*.

An autoecious life cycle for *T. areolata* through aeciospore re‐infections on *Picea* has been suggested as one possible explanation for the repeated infections found in isolated seed orchards with no telial hosts within or in close proximity of the plantation (Kaitera, 2013; Kaitera et al., 2014; Kaitera & Tillman‐Sutela, 2014). Such autoecious replication should result in identical fungal genotypes spread among cones like in the case of the autoecious rust, *P. pini* (Hantula et al., 1998; Samils et al., 2011). Our data support that *T. areolata* is a macrocyclic heteroecious rust, which utilizes two host plants and all five spore stages as suggested earlier (Sato & Takahashi, 1970).

Actually, it has been reported that aeciospores can germinate in young shoots without forming other structures (Kuprevich & Tranzschel, 1957), but this seems to be a rare finding since results of artificial inoculations have been negative (pers. comm.) and the biological mechanisms how aeciospores could colonize cones are unknown. However, there is evidence of colonization of *Picea* shoots by *T. areolata* as reported by Hietala, Solheim, and Fossdal (2008) who found the fungus in 100 symptomatic young seedlings of *P. abies* by quantitative PCR (Hietala et al., 2008). Among other rusts, the autoecious stem rust on Scots pine, *P. pini*, is able to infect the host by aeciospores, grow and sporulate from spermogonia and aecia in host tissues systematically for years (Kaitera & Nuorteva, 2008). In the case of *P. pini,* it is unknown, if spermogonia are nonfunctional or the spermatization takes place by self‐fertilization. If autoecism exists in *T. areolata*, it is probably rare and insignificant or occurs at low frequency. In the case of *T. areolata,* successful inoculations on the host by aeciospores could confirm autoecism of the rust. Much biological and genetic evidence is still needed to fully understand the mode of reproduction of *T. areolata* in *P. abies*.

## 5 CONCLUSIONS

In this study, we have clarified several parts of the life cycle of the fungi *T. areolata*, the causal agent for cherry‐spruce rust, which is a serious problem in Norway spruce seed orchards. *Thekopsora areolata* has a heteroecious life cycle, and the majority of infections of spruce cones are a result from basidiospore infections and mating. The main objective of this study was to investigate the population genetics and reproductive biology of the rust fungus *T. areolata*. The analysis together with a hierarchical sampling showed apparent random mating and common sexual events. Moreover, it showed that the rust populations in the locations sampled belonged to the same metapopulation with no obvious genetic structure. Additionally, it highlighted the complex mixed mode of reproduction of *T. areolata* in *P. abies* cones with multiple infections and vegetative spread in the cone and scale after fertilization. Overall, these findings showed high genotypic diversity and high levels of gene flow in *T. areolata* in Fennoscandia. Although no genetic differentiation is found between locations, probably due to long‐distance spread by aecio‐ or urediniospores, local disease pressure is most likely supported by *P. padus* trees in close proximity of the seed orchards and should be eradicated if possible.

## CONFLICT OF INTEREST

Authors declare no conflict of interest.

## AUTHOR CONTRIBUTIONS

**Hernán Capador:** Conceptualization (supporting); data curation (lead); formal analysis (lead); investigation (supporting); methodology (supporting); project administration (supporting); validation (equal); visualization (lead); writing–original draft (equal); writing–review and editing (supporting). **Berit Samils:** Conceptualization (equal); data curation (supporting); formal analysis (supporting); funding acquisition (supporting); investigation (supporting); methodology (equal); project administration (supporting); software (equal); supervision (equal); validation (equal); writing–original draft (supporting); writing–review and editing (supporting). **Juha Kaitera:** Conceptualization (supporting); investigation (equal); project administration (supporting); supervision (supporting); validation (supporting); writing–original draft (supporting); writing–review and editing (supporting). **Åke Olson:** Conceptualization (lead); data curation (supporting); formal analysis (supporting); funding acquisition (lead); investigation (equal); methodology (equal); project administration (lead); resources (equal); supervision (lead); validation (supporting); visualization (supporting); writing–original draft (lead); writing–review and editing (lead).

## Supporting information

Figure S1

Table S1

## Data Availability

Information on sample location and microsatellite genotypes are available at Dryad https://doi.org/10.5061/dryad.2ngf1vhk9
