# Supplementary material for: Genetic evidence for sexual reproduction and multiple infections of Norway spruce cones by the rust fungus Thekopsora areolata
Source: Ecol Evol. 2020 Jun 17;10(14):7389–403. doi: 10.1002/ece3.6466 (PMC7391340; doi:10.1002/ece3.6466)
Supplement: Supplementary file 1 — Figure S1 [file ECE3-10-7389-s001.pdf]

Supporting Information Figure S1

K=2

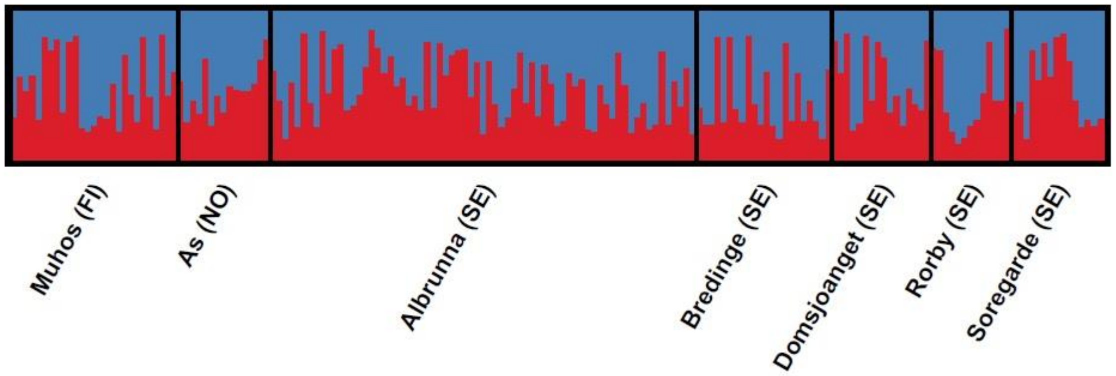

K=3

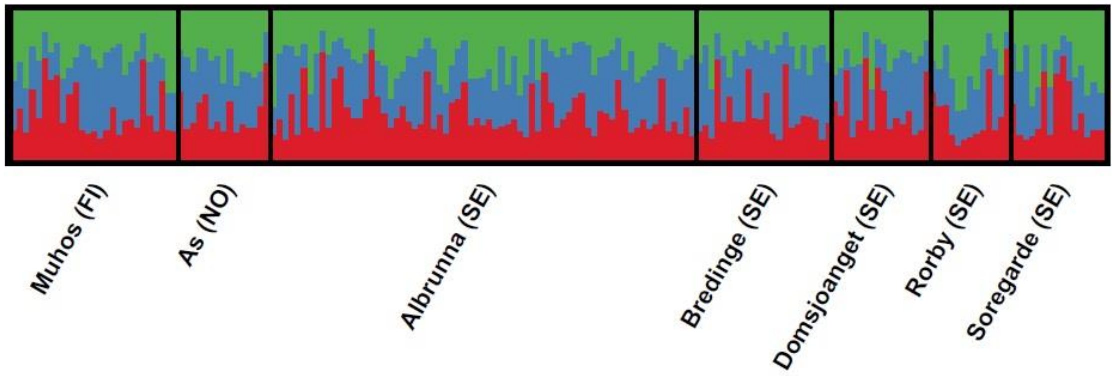

K=4

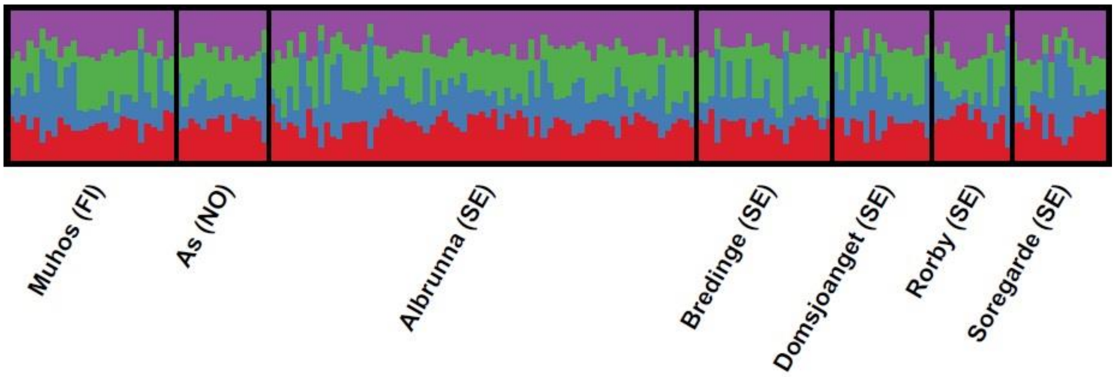

K=5

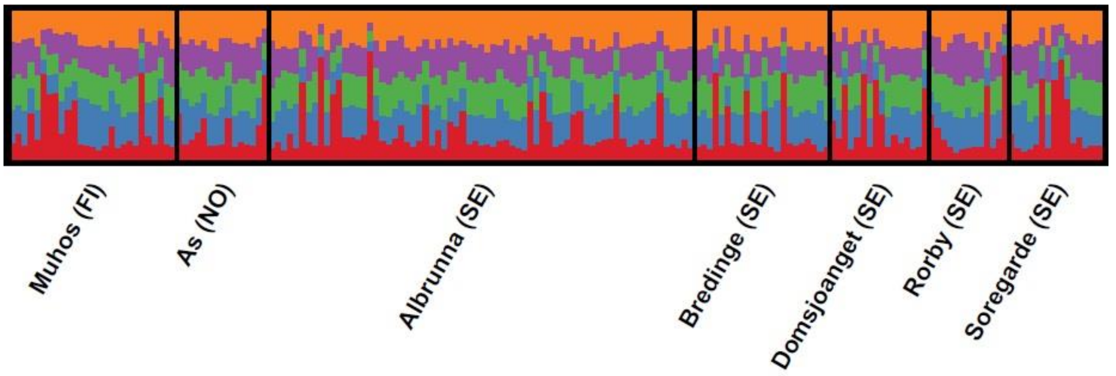

Figure S1. Bayesian cluster analysis using STRUCTURE software for 177 individuals of *Thekopsora areolata* from 7 seed orchards in Finland (FI), Norway (NO) and Sweden (SE). Bar plots for the assignment of the individuals to 2, 3, 4 and 5 clusters (K) are shown.
