# Supplementary material for: Genetic evidence for sexual reproduction and multiple infections of Norway spruce cones by the rust fungus Thekopsora areolata
Source: Ecol Evol. 2020 Jun 17;10(14):7389–403. doi: 10.1002/ece3.6466 (PMC7391340; doi:10.1002/ece3.6466)
Supplement: Supplementary file 2 — Table S1 [file ECE3-10-7389-s002.docx]

**Supplementary Information**

**Table S1.** Pairwise Population Matrix of Fst Values for *Thekopsora areolata*

| Populations | SE_AL | SE_BR | SE_RO | SE_SO | SE_DO | NO_AS | FI_MU |
| --- | --- | --- | --- | --- | --- | --- | --- |
| SE_AL | 0.000 | 0.048 | 0.303 | 0.436 | 0.145 | 0.371 | 0.161 |
| SE_BR | 0.026 | 0.000 | 0.111 | 0.263 | 0.303 | 0.263 | 0.111 |
| SE_RO | 0.022 | 0.032 | 0.000 | 0.679 | 0.303 | 0.182 | 0.223 |
| SE_SO | 0.018 | 0.022 | 0.019 | 0.000 | 0.835 | 0.785 | 0.526 |
| SE_DO | 0.029 | 0.023 | 0.029 | 0.015 | 0.000 | 0.271 | 0.335 |
| NO_AS | 0.024 | 0.028 | 0.036 | 0.019 | 0.032 | 0.000 | 0.537 |
| FI_MU | 0.017 | 0.020 | 0.023 | 0.016 | 0.021 | 0.020 | 0.000 |
| Fst values below the diagonal. Probability, P(rand >= data) based on 999 permutations is shown above diagonal. | | | | | | | |
